# Joint Profiling of miRNAs and mRNAs Reveals miRNA Mediated Gene Regulation in the Göttingen Minipig Obesity Model

**DOI:** 10.1371/journal.pone.0167285

**Published:** 2016-11-30

**Authors:** Caroline M. Junker Mentzel, Ferhat Alkan, Helle Keinicke, Mette J. Jacobsen, Jan Gorodkin, Merete Fredholm, Susanna Cirera

**Affiliations:** 1 Animal Genetics, Department of Veterinary Clinical and Animal Science, Faculty of Health Sciences, University of Copenhagen, Frederiksberg, Denmark; 2 Center for non-coding RNA in Technology and Health, Computational Biology and Bioinformatics, Department of Veterinary Clinical and Animal Science, Faculty of Health Sciences, University of Copenhagen, Frederiksberg, Denmark; Kunming University of Science and Technology, CHINA

## Abstract

Obesity and its comorbidities are an increasing challenge for both affected individuals and health care systems, worldwide. In obese individuals, perturbation of expression of both protein-coding genes and microRNAs (miRNA) are seen in obesity-relevant tissues (i.e. adipose tissue, liver and skeletal muscle). miRNAs are small non-coding RNA molecules which have important regulatory roles in a wide range of biological processes, including obesity. Rodents are widely used animal models for human diseases including obesity. However, not all research is applicable for human health or diseases. In contrast, pigs are emerging as an excellent animal model for obesity studies, due to their similarities in their metabolism, their digestive tract and their genetics, when compared to humans. The Göttingen minipig is a small sized easy-to-handle pig breed which has been extensively used for modeling human obesity, due to its capacity to develop severe obesity when fed *ad libitum*. The aim of this study was to identify differentially expressed of protein-coding genes and miRNAs in a Göttingen minipig obesity model. Liver, skeletal muscle and abdominal adipose tissue were sampled from 7 lean and 7 obese minipigs. Differential gene expression was investigated using high-throughput quantitative real-time PCR (qPCR) on 90 mRNAs and 72 miRNAs. The results revealed de-regulation of several obesity and inflammation-relevant protein-coding genes and miRNAs in all tissues examined. Many genes that are known to be de-regulated in obese humans were confirmed in the obese minipigs and several of these genes have target sites for miRNAs expressed in the opposing direction of the gene, confirming miRNA-mediated regulation in obesity. These results confirm the translational value of the pig for human obesity studies.

## Introduction

Obesity is an increasing problem in the developed world due to obesity derived co-morbidities, such as dyslipidemia, type 2 diabetes, high blood pressure, cardio vascular disease and cancer, which are life threatening and costly for health care systems [1]. Obesity is defined as an excess in accumulation of adipose tissue. Adipose tissue is an endocrine organ and many of the obesity-derived comorbidities are linked to dysfunctional adipose tissue. Signaling in adipose tissues occur to and from other tissues such as the skeletal muscle and liver, which are affected by lipid spill-over from the adipose tissue and from the chronic low grade inflammation commonly seen in obesity [2]. Changes in adipose tissue signaling can be measured at the RNA level and many of the obesity-relevant genes are potentially regulated by microRNAs (miRNAs). miRNAs are small non-coding RNAs that bind predominantly to the 3’untranslated region (3‘UTR) of target mRNAs and degrade the mRNAs and/or inhibit their translation. miRNAs regulate genes in many physiological processes, as well as in different developmental stages and in different disease states, including obesity [3].

Pigs are an excellent model for human diseases due to their similarities in physiology, organ size, genetics and metabolism [4,5]. The Göttingen minipig is a frequently used model for human diseases due to their smaller size compared to production pigs, which makes housing and handling of the pigs easier. Furthermore, Göttingen minipigs spontaneously develop obesity when fed *ad libitum* [6,7]. Female Göttingen minipigs have the potential to become more obese, more insulin-resistant and have higher plasma lipid levels than male Göttingen minipigs [8]. Recent findings from our group examening quantitative trait locis (QTLs) influencing obesity and metabolic traits in a Göttingen minipig x production pig crossbreed obesity model, revealed that several genes located in obesity-relevant QTLs, overlap with findings in human studies [9]. This research emphasizes the value of a porcine model for human obesity. Moreover, in our previous research we have shown that miRNAs are differentially expressed in lean versus obese pigs from the same pig population, confirming their relevance in obesity studies [10]. One challenge with working with the pig as a model for miRNA studies is the issue of miRNA target finding. *In silico* miRNA target finding websites and databases of experimentally supported miRNA targets, are only available for human and some other common model organisms such as the mouse and the worm [11–15]. Hence, custom target finding strategies must be designed for miRNA target finding in pigs.

Studying the expression of protein-coding genes and miRNAs in obesity-relevant tissues of obese and lean animals, may reveal some of the underlying genetic and regulatory mechanisms in the progression of obesity and may further validate the Göttingen minipig as good model for obesity. In this study, gene and miRNA expression was investigated in the liver, skeletal muscle and abdominal adipose tissue of 7 obese and 7 lean female minipigs. A custom strategy for finding miRNA-targets within differentially expressed protein coding genes was also applied.

## Materials and Methods

### Animal material

The 14 female ovariectomized Göttingen minipigs used for the study were housed at the animal research facilities at the University of Copenhagen (Taastrup, Copenhagen) and humanely euthanized by pentobarbital injection followed by bleeding at 41–47 months of age. The lean group (n = 7), were fed restrictively 150 g of standard minipig chow, two times a day and the obese group (n = 7), were fed *ad libitum*. The obese group had previously been used in pharmacological studies with therapeutic peptides but underwent a suitable wash-out period, based on multiple half-lives of the peptide, prior to this study. Abdominal (retroperitoneal) adipose tissue, skeletal muscle and liver were collected immediately after euthanization, snap-frozen in liquid nitrogen and stored at -80°C. The Danish Animal Experiments Inspectorate approved all experimental procedures involving the Göttingen minipigs Animal care and maintenance was performed according to the Danish “Animal Maintenance Act”, Act 432 dated 09/06/2004.

### RNA purification

Frozen tissue was homogenized in Tri Reagent (MRC gene, Molecular Research Center, Inc) using a gentleMACS Octo Dissociator (Miltenyi Biotec). RNA extraction was performed according to the manufacturer’s protocol, using chloroform for phase separation. For RNA extraction from adipose tissue, visible fat was removed following the first centrifugation step, before proceeding with the protocol.

Liver and muscle RNA samples to be used for mRNA qPCR were DNAse treated using DNA-freeTM Kit (Ambion) with a reaction volume of 50 μl and a maximum RNA content of 10 μg, according to the protocol. Abdominal adipose tissues samples were not DNase treated, due to very low RNA yields.

RNA concentration was measured using a NanoDrop ND-1000 Spectrophotometer (Thermo Fisher Scientific) and the purity assessed from the OD 260/280 ratio.

RNA integrity was measured by the Experion system (Bio-Rad) using the Eukaryote Total RNA StdSens Analysis Kit. Only samples with a RQI ≥ 7 (6 for abdominal fat) were used for downstream analysis (Mean ± SD of RQI: Abdominal adipose tissue 7.90 ± 0.64, liver 8.54 ± 0.64 and skeletal muscle 7.98 ± 0.67).

### cDNA synthesis

cDNA synthesis for the mRNA qPCR study was made in duplicates from 200 ng RNA of each sample in a final reaction volume of 10 μl. 0.5 μl Improm-II^™^ reverse transcriptase (Promega), 0.25 μg 1:3 OligodT/random primers, 2 μl ImProm-II buffer, 10 units RNasin Ribonuclease inhibitor (Promega), 2.5 mM MgCl2 and 2 mM dNTP mix. Reactions were incubated for 5 min at room temperature, 1 hour at 42°C and 15 min at 70°C to inactive the enzyme according to the manufacturer’s instructions. A negative control was made for each tissue with no reverse transcriptase added (-RT control). The cDNA was diluted 1:8 prior to qPCR and stored at -80°C until use.

cDNA synthesis for the miRNA qPCR study was made according to the miRspecific method [16,17]. Briefly, cDNA was made in duplicates from 100 ng RNA of each samples in a final reaction volume of 10μl. 1μl 10x poly(A) polymerase buffer ((New England Biolabs), 0.1 mM ATP, 1 μM RT-primer (5’-CAGGTCCACTTTTTTTTTTTTTTTVN; V = A, C and G; N = A, C, G and T, TAG Copenhagen), 0.1 μM dATP, 0.1 μM dCTP, 0.1 μM dGTP, 0.1 μM dTTP, 100 units MuLV reverse transcriptase (New England Biolabs) and 1 unit poly(A) polymerase (New England Biolabs). The cDNA was diluted 1:16 prior to qPCR and stored at -80°C until use.

### Primer design

The genes included in this study were individually selected based on results from previous obesity studies in pigs and other organisms as well as unpublished information obtained from conferences [9,10,18–20]. Primer sequences for protein coding gene expression were designed using the Primer3 software (http://bioinfo.ut.ee/primer3/). They were designed to make a product in the range of 75–200 nucleotides, and if possible, were designed to span a large intron. Some primer sequences were also obtained from other pig studies [21–23].

Primers for miRNA were designed using the miRprimer software [24]. All miRNA primer sequences have previously been published [18]. All primers sequences can be found in the supplementary file S1 Table.

### qPCR

High-Throughput qPCR was conducted using the Biomark HD system (Fluidigm Corporation) on a 96.96 IFC chip. 15 cycles of pre-amplification of 8x diluted cDNA using TaqMan PreAmp Master Mix (Life Technologies) and subsequent cleanup with Exonuclease I (New England BioLabs) was performed according to the manufacturer’s protocol (Fluidigm PN 100–5875 C1). A single modification was made, including an altered concentration of primers at 250 nM in the primer pool. Exonuclease cleaned cDNA was diluted 5x before running the qPCR reactions using SsoFastTM EvaGreen^®^ Supermix with Low ROX (Bio-Rad Laboratories) according to the manufacturer’s instructions (PN 100–9792 B1) with a modification of using primer concentrations of 5 μM. Standard curves were performed using pre-amplified cDNA in 5x dilution rows. Data was obtained using the associated software.

### qPCR data analysis

The efficiency of the primer assays was calculated from the log-linear portion of the standard curves. For high-throughput qPCR efficiency of 85–110% was accepted with an R^2^ > 0,98. miRNAs that had expression levels outside the standard curve, but had robust data had their efficiencies set to 1. All qPCR data was analyzed using GenEx6 Pro (MultiD Analyses AB).

For the protein-coding gene expression study, quantification cycle (Cq) values where normalized to the geometric mean of the most stable assays (Adipose tissue: 45 genes, Liver: 38 genes, Muscle: 54 genes) which were determined using the NormFinder algorithm [25]. miRNA qPCR data was normalized to the mean expression value of all expressed miRNAs as recommended for large-scale miRNA studies [26].

Technical replicates from the reverse transcription were averaged. Relative expression of the lowest expressed sample for each assay was set to 1 and the data was log2 transformed to achieve normal distribution. Student’s t-test was used for statistical analysis. Due to limited material from the abdominal adipose tissue samples, only 5 animals in the obese group was used for the miRNA study. Raw Cq values, efficiencies and t-test results are shown in the supplementary file, S1 Dataset.

All figures were produced using Graphpad Prism 6 (Graphpad Software).

### miRNA-target interactions

To the best of our knowledge, no compiled database reporting miRNA binding sites in porcine transcripts exists. Therefore, two parallel strategies were used to find the links between differentially regulated miRNAs and mRNAs; 1) miRNA target prediction using pig transcript sequences and 2) homologous interaction search using human transcripts.

Strategy 1: The reference sequence (refSeq) transcript identifiers of the sequences targeted by the primers was confirmed and retrieved by the Primer-BLAST web interface [27]. mRNA sequences in FASTA format were manually retrieved using the NCBI web interface and open reading frame (ORF) region information was used to locate the 3’UTR regions. Mature miRNA sequences were obtained from miRBase version 21 [28]. Three different tools, miRanda [29], PITA [30], and RIsearch2 (a suffix array enhanced improved version of RIsearch) (Alkan *et al*, submitted) [31], were used to predict miRNA-mRNA interactions between differentially expressed pig transcripts using default parameter settings. This was performed including both the differentially expressed mature miRNA and full mRNA sequences. In this study, interactions predicted on ORF and 5’UTR regions were filtered out and we only focused on the canonical miRNA target sites within 3’UTR regions.

Strategy 2: interactions between homologous miRNA and mRNA sequences in human were also assessed. Homologous mature miRNA sequences in humans were identified using the “search by sequence” option in miRBase using the mature miRNA sequences of all differentially expressed pig miRNAs. For differentially expressed pig mRNAs, homologous protein-coding genes were retrieved from Ensembl version 84 by using BioMart martview [32]. To find experimental support for interactions between identified human miRNA and mRNA homologs, TarBase v7.0 [11] was queried for homologous miRNA-mRNA pairs with experimental support for interaction. The RAIN database (http://rth.dk/resources/rain), which incorporates RNA interactions into the STRING database (Junge *et al*, submitted) [33] was also queried to find further experimental evidence for possible RNA–RNA interactions within human homologs. The RAIN database compiles RNA–RNA interactions, not only from various experimental miRNA target databases, such as StarBase [12] and miRTarBase [13], but also provides interaction predictions between all human miRNAs and 3’UTR sequences by using several different tools, including miRanda, PITA, TargetScan [14] and STarMiRDB [15].

All interactions can be found in S2 Dataset.

## Results

Lean and obese minipigs differ significantly in their bodyweight (kg±SEM, 50.3±1.6 vs. 92.6±5.2) and other physical traits, such as fat mass measured by dexa scan (kg±SEM. 12.9±1.0 vs. 36.4±2.7). However, they do not differ significantly in their plasma lipid levels. A full table of phenotypic traits registered in these minipigs has been previously published [6].

**Fig 1:**
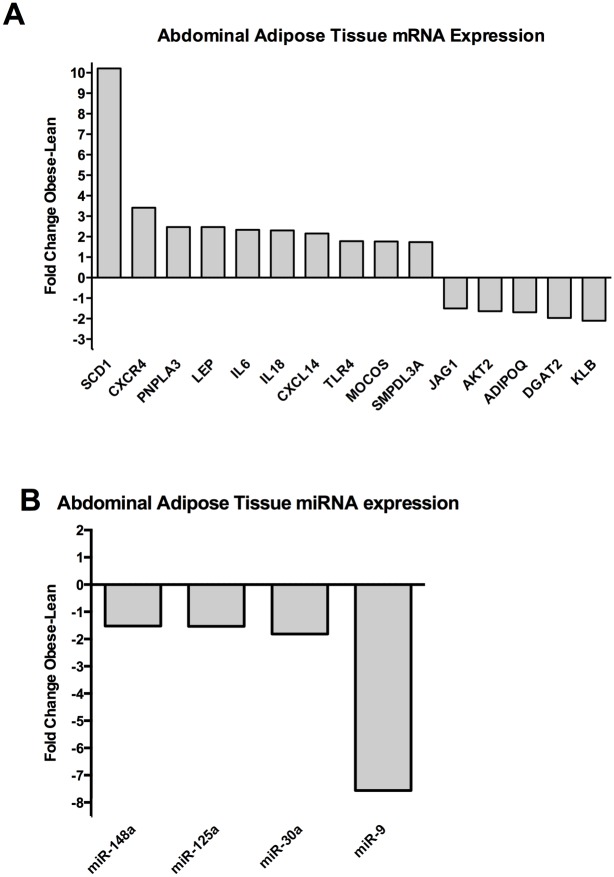
Expression of protein coding genes and miRNAs in adipose tissue. (A) Protein-coding genes and (B) miRNAs with a fold change of > ±1.5 and significant differential expression with a p value < 0.05 (Student´s t test) are shown. The fold change (Obese/Lean) for each significant gene is shown. A positive fold change denotes upregulation in obese Göttingen minipigs and a negative fold change denotes down regulation in obese Göttingen minipigs.

**Fig 2:**
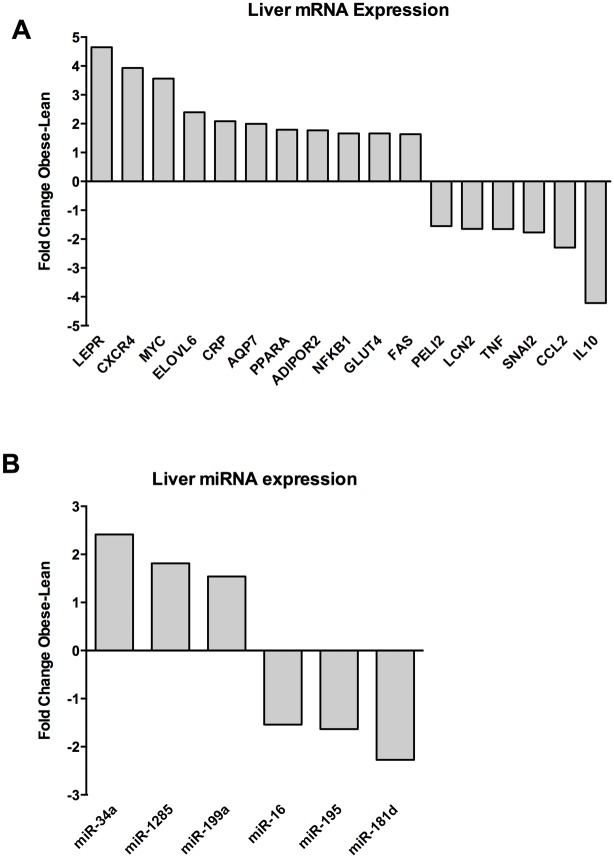
Expression of protein coding genes and miRNAs in liver. A) Protein-coding genes and (B) miRNAs with a fold change of > ±1.5 and significant differential expression with a p value < 0.05 (Student´s t test) are shown. The fold change (Obese/Lean) for each significant gene is shown. A positive fold change denotes upregulation in obese Göttingen minipigs and a negative fold change denotes downregulation in obese Göttingen minipigs.

**Fig 3:**
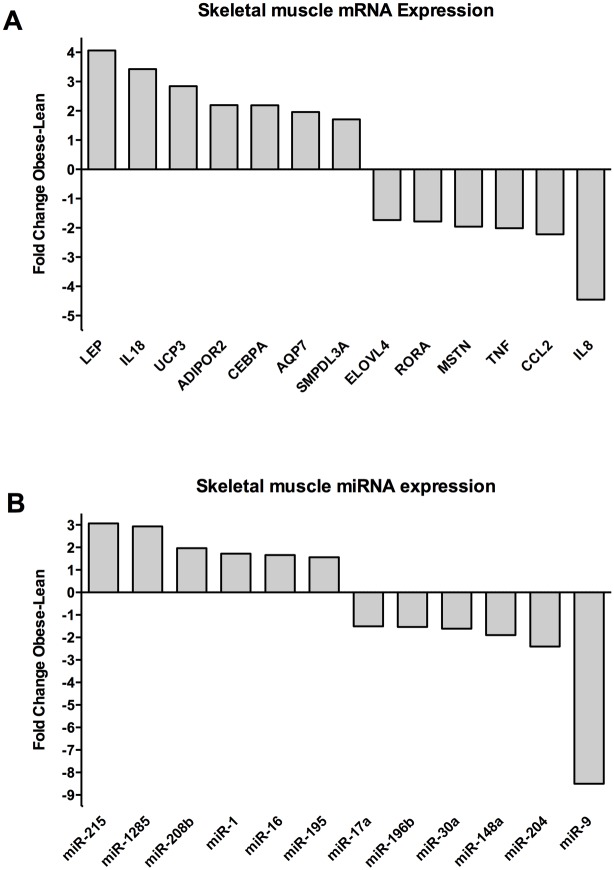
Expression of protein coding genes and miRNAs in skeletal muscle. Protein coding genes (A) and miRNA (B) with a fold change of > ±1.5 and significant differential expression with a p value < 0.05 (Student´s t test) are shown. The fold change (Obese/Lean) for each significant gene is shown. A positive fold change denotes upregulation in obese Göttingen minipigs and a negative denotes downregulation in obese Göttingen minipigs.

High-throughput qPCR on both lean and obese Göttingen minipigs showed significantly different expression profiles in all three tissues for both protein-coding genes and miRNAs. Figs 1–3 shows all significantly differently (p < 0.05, t-test) differentially expressed genes and miRNAs with a fold change (FC) of either above or below 1.5, depending on up- or downregulation in the obese minipigs. The genes with the highest fold change are reported for each individual tissue with p values in the sections below. Raw Cq values and t-test data for all analyzed genes and miRNAs are reported in supplementary file S1 Dataset.

### Differentially expressed protein-coding genes and miRNAs in abdominal adipose tissue

The differentially expressed protein-coding genes are shown in Fig 1A. Stearoyl-CoA Desaturase (*SCD1*) (FC 10.2; p value: 2.71x10^-4^) was the most upregulated gene in the obese pigs and the second most upregulated gene was Chemokine (C-X-C Motif) Receptor 4 (*CXCR4*) (FC 3.4; p value: 6.23x10^-5^). Patatin-Like Phospholipase Domain Containing 3 (*PNPLA3*), Leptin (*LEP*), Interleukin 6 (*IL-6*), Interleukin 18 (*IL-18*), Chemokine (C-X-C Motif) Ligand 14 (*CXCL14*), Toll-Like Receptor 4 (*TLR4*), Molybdenum Cofactor Sulfurase (*MOCOS*) and Sphingomyelin Phosphodiesterase, Acid-Like 3A (*SMPDL3A*) were all upregulated and had fold changes > 1.5 and p values < 0.05. The most downregulated gene was β-klotho (*KLB*) (FC 2.1; p value: 1.81x10^-5^). Diacylglycerol O-Acyltransferase 2 (*DGAT2*), Adiponectin (*ADIPOQ*), V-Akt Murine Thymoma Viral Oncogene Homolog 2 (*AKT2*) and Jagged 1 (*JAG1*) were all downregulated with fold changes of < -1.5 and p values < 0.05.

Differentially expressed miRNAs are shown in Fig 1B. No miRNAs were upregulated with a fold change > 1.5 and a p value < 0.05. However, miR-9 was highly downregulated (FC 7.6; p value 0.046). In addition, miR-30a, miR-125a and miR-148a all had fold changes of < -1.5 and p values < 0.05.

### Differentially expressed protein-coding genes and miRNAs in liver

Differentially expressed protein-coding genes are shown in Fig 2B. Leptin receptor (*LEPR*) (FC 4.6; p value: 0.03), *CXCR4* (FC 3.93; p value: 2.31x10^-5^) and v-Myc Avian Myelocytomatosis Viral Oncogene homolog *(MYC*) (FC 3.56; p value: 0.03) were the most highly upregulated genes in obese minipig liver and Interleukin 10 (*IL-10*) and Chemokine (C-C Motif) Ligand 2 Homolog (*CCL2*) were the most downregulated. ELOVL Fatty Acid Elongase 6 (*ELOVL6*), C-reactive protein (*CRP*), Aquaporin 7 (*AQP7*), Peroxisome proliferator-activated receptor A (PPARA), Adiponectin Receptor 2 (*ADIPOR2*), Nuclear Factor Of Kappa Light Polypeptide Gene Enhancer In B-Cells 1 (*NFKB1*), Glucose transporter 4 (*GLUT4*) and Tumor Necrosis Factor Receptor Superfamily, Member 6 (*FAS*) were all up regulated with fold changes of > 1.5 and p values < 0.05. Snail Family Zinc Finger 2 (*SNAI2*), tumor necrosis factor A (*TNF*), Lipocalin 2 (*LCN2*) and Pellino E3 Ubiquitin Protein Ligase Family Member 2 (*PELI2*) were all downregulated with fold changes of < -1.5 and p values < 0.05.

Differentially expressed miRNAs are shown in Fig 2B. MiR-34a (FC 2.4; p value 1.0x10^-3^) was the most upregulated miRNA. MiR-1285 and miR-199a-5p were also up regulated with fold changes of > 1.5 and p values < 0.05. MiR-181d (FC 2.27; p value 0.03) was the most downregulated while miR-195 and miR-16 were down regulated with fold changes < -1.5 and p values < 0.05.

### Differentially expressed protein coding genes and miRNAs in skeletal muscle

Differentially expressed protein-coding genes are shown in Fig 3A. *LEP* (FC 4.1; p value 0.027), *IL-18* (FC 3.4, p value 1.2x10^-3^) and Uncoupling Protein 3 (*UCP3*) (FC 2.8; p value 1.7x10^-3^) were the most upregulated genes in muscle. *ADIPOR2*, CCAAT/Enhancer Binding Protein a (*C/EBP-a*), *AQP7* and *SMPDL3A* were all up regulated with fold changes of > 1.5 and p values < 0.05. IL8 (FC -4.5; p value 0.01) was the most down regulated gene. *CCL2*, *TNFa*, Myostatin (*MSTN*), Retinoic acid receptor-related orphan receptor α (*RORA*) and ELOVL Fatty Acid Elongase 4 (*ELOVL4*) were all downregulated with fold changes of < -1.5 and p values < 0.05.

Differentially expressed miRNAs are shown in Fig 3B. MiR-215-5p (FC 3.1; p value 0.02) and miR-1285 (FC 2.9; p value 7.35x10^-5^) were the most upregulated miRNAs. MiR-208b-3p, miR-1, miR-16 and miR-195 were upregulated with a fold change of > 1.5 and p value < 0.05. MiR-9 (FC -8.5; p value 0.02) was the most downregulated miRNA. MiR-204, miR-148a, miR-30a, miR-196b, and miR-17a were downregulated with fold changes of < -1.5 and p values < 0.05.

### miRNA-target analysis

The results from the miRNA-target analysis are summarized in supplementary file S2 Dataset, where predicted interactions between differentially expressed pig miRNA and pig mRNAs can be observed together with predicted and experimental support in human homologs for interactions.

Table 1 shows selected predicted miRNA-mRNA interactions. miRNA-mRNAs were selected if PITA, miRanda and RIsearch2 all showed potential interactions in pig, or if two of them showed potential interactions in pig, together with supplementary experimental evidence in human. A couple of interactions are shown where there is extensive support for interaction in humans, but none in pigs. *LEP* was the gene containing the most miRNA target sites, i.e. is targeted by miR-148a-3p, miR-125a-5p, miR-30a, miR-9-5p and miR-17-5p. All these miRNA-target interactions have support from both porcine and human predictions. *SCD* is also targeted by many of the same miRNAs, namely miR-148a-3p, miR-125a-5 and miR-9-5p.

**Table 1:** miRNA-target interactions.

Tissue	miRNA	mRNA	Target in pig	Target in human
**Adipose tissue**	miR-148a-3p	*LEP*	P, M, R	P
		*SCD*	P, M, R	M, Exp
	miR-125a-5p	*LEP*	P, M, R	P, M, T
		*SCD*	P, M, R	P, M, T, Exp
	miR-30a	*PNPLA3*	P, M, R	-
		*LEP*	P, M, R	P,
	miR-9-5p	*LEP*	P, M, R	P, M, T
		*SCD*	P, M, R	P, M, T
		*CXCR4*	-	P, D, M, T, Pi
		*CXCL14*	P, M, R	P
**Liver**	miR-34a	*LCN2*	P, M, R	Exp
	miR-1285	*LCN2*	P, M, R	-
	miR-181d-5p	*ADIPOR2*	P, R	P, Exp
		*FAS*	P, M, R	P, M, Exp
		*PPARA*	-	T, Exp
	miR-16	*GLUT4*	P, R	Exp
		*FAS*	P, M, R	-
		*NFKB1*	P, M, R	-
	miR-195-5p	*FAS*	P, M, R	-
**Muscle**	miR-1	*RORA*	P, M, R	-
		*TNF*	P, M, R	-
	miR-195-5p	*RORA*	P, M, R	-
	miR-16	*CCL2*	-	Exp
		*RORA*	P, M, R	-
		*ELOVL4*	P, M, R	P, M, S
	miR-30a	*LEP*	P, M, R	P,
		*UCP3*	P, M, R	P, M, T
	miR-9-5p	*CEBPA*	P, M, R	-
		*LEP*	P, M, R	P, M, T
		*UCP3*	P, M, R	P, M, T
	miR-204	*CEBPA*	P, R	Exp
		*ADIPOR2*	P, R	P, M, Exp
	miR-196b-5p	*ADIPOR2*	P, R	Exp
	miR-148a	*LEP*	P, M, R	P
	miR-17-5p	*LEP*	P, M, R	P,M
		*UCP3*	P, M, R	P, M, S, T

miRNA and target genes with support for a miRNA-target site by 3 tools in pig or experimental evidence in humans. The full list of interactions is available in Supplementary file S2 Dataset. P: PITA, M: miRanda, R: RIsearch2 T: TargetScan, D: miRDB, Pi: Pictar, S: STarMiRDB, Exp: Experimental evidence. miRNA annotation follows the miRBase annotation for porcine miRNAs.

## Discussion

In this study, 7 lean and 7 obese Göttingen minipigs were used for studying differential mRNA and miRNA expression in abdominal adipose tissue, liver and skeletal muscle. In total, 40 protein-coding genes and 18 miRNAs were significantly differentially expressed with fold changes larger than 1.5 suggesting important differences in obesity-relevant gene expression between lean and obese Göttingen minipigs. We expected to encounter a large number of differentially expressed protein-coding genes and miRNAs in this study, since the genes included were individually selected, based on results from previous obesity studies in pigs and other organisms as well as unpublished information obtained from conferences [9,10,18–20]. Previous studies using the same animals have shown significant differential expression of several other inflammation (mainly genes involved in innate immunity) and obesity relevant genes in multiple tissues as well as a slight enlargement of the adipocytes within the obese minipigs [20,22]. A custom miRNA-target finding strategy was applied and miRNA-target sites were discovered in several of the differentially expressed genes.

Many of the differentially expressed genes in obese versus lean minipigs were de-regulated in the same pattern as seen in studies of obese human and mice. Furthermore, several of these genes were targeted by miRNAs previously detected in obesity studies. *IL18*, *LEP* and *SMPDL3A* were upregulated in both adipose tissue and muscle of obese pigs and the leptin receptor, *LEPR*, was upregulated in the obese liver. IL-18 is a pro-inflammatory cytokine expressed in macrophages but also adipocytes and muscle cells and its expression correlates with obesity, type 2 diabetes and the metabolic syndrome [34]. Serum leptin levels correlates with obesity and hepatic steatosis and the leptin receptor in liver regulates lipid droplet accumulation in the liver [35,36]. *LEP* has target sites for three miRNAs: MiR-30a, miR-148a and miR-9-5p which were all downregulated in obese adipose tissue and muscle. MiR-30a and miR-148 are both involved in adipocyte differentiation, downregulated in obese adipose tissue in mice and are involved in myogenic differentiation [37–39]. In contrast, MiR-9-5p is upregulated in serum of human diabetic patients and, in another study, upregulated in porcine adipose tissue from a mixed breed population [10,40]. *LEP* also has a target site for miR-125a, which is downregulated in obese adipose tissue, an observation in agreement with human studies [41]. MiR-9-5p, miR-148a and miR-125a also have target sites in *SCD*, which is upregulated in the adipose tissue of the obese minipigs. SCD is a catalyzer of fatty acid conversion and the transcript is also upregulated in obese rats and in humans where *SCD* expression in adipose tissue correlates with BMI [42,43]. *SMPDL3A* is upregulated by cholesterol loading in human macrophages and is upregulated in thoracic aorta of obese Ossabaw pigs and in adipose tissue of obese pigs [19,44,45].

*PNPLA3*, *IL-6*, *CXCL14* and *TLR4* are upregulated in the adipose tissue of obese minipigs. *PNPLA3* has a target site for miR-30a. *PNPLA3* is upregulated in adipose tissue of obese mice and its expression is regulated by changes in the energy balance in humans [46,47]. IL-6 is a pro-inflammatory cytokine that regulates lipid metabolism in adipose tissue and plasma IL-6 levels correlates with BMI in humans [48,49]. *CXCL14* encodes a macrophage chemo-attractant and is also upregulated in adipose tissue of obese mice [50]. *CXCL14* has a target site for miR-9-5p. TLR4 activates pro-inflammatory responses and is upregulated in adipocytes of obese mice [51].

*ELOVL6*, *CRP* and *FAS* are all upregulated in the obese liver. ELOVL6 promotes development of non-alcoholic steatohepatitis (NASH) and insulin resistance [52,53]. CRP levels in blood are used to measure inflammation, but is also elevated in liver of obese humans, independently if they suffer from metabolic syndrome and NASH [54]. FAS is a cell surface death receptor involved in apoptosis and it’s expression is increased in patients with NASH [55]. *FAS* has target sites for miR-195, miR-181d and miR-16 that were all downregulated in the liver of the obese minipigs. In contrast, miR-195 is upregulated in the liver of type 2 diabetic rats [56].

*CEBPA* and *UCP3* are upregulated in the obese muscle. C/EBP-α is a transcription factor involved in macrophage activation [57]. It has target sites for miR-9-5 and miR-204. UCP3 facilitates fatty acid uptake and metabolism and overexpression of *UCP3* lowers intramuscular triglyceride content in mice [58]. If mice are fed a high fat diet *UCP3* is upregulated and gene variants for *UCP3* are associated with childhood obesity [59,60]. Furthermore UCP3 has a target site for miR-17a which is also down regulated in human obesity [61].

Some genes, which have been shown to have a protective function when overexpressed, are up regulated in the obese minipigs, but are normally down regulated in obesity in humans or rodents. One example is *CXCR4* which is upregulated in liver and adipose tissue. CXCR4 is a chemokine receptor expressed in adipocytes, macrophages and hepatic stellate cells that protects against obesity and obesity associated inflammation [62,63]. In humans, *CXCR4* is a verified target gene of miR-9 [64] and there is extensive support for a human target site in Table 1, but no support for a porcine target site. Another example of protective gene expression is *ADIPOR2* which is upregulated in both obese liver and muscle. Overexpression of *ADIPOR2* in the liver of NASH model mice showed that *ADIPOR2* expression in liver improves NASH [65]. *ADIPOR2* is targeted by the downregulated miRNAs miR-181d, miR-92a, miR-204 and miR-196b-5p. MiR-181d is downregulated in serum of NAFLD patients [66]. MiR-92a is generally downregulated in animal models of diabetes [67]. MiR-204 is also downregulated in adipose tissue of obese mice [68].

Some obesity and/or pro-inflammatory genes are also down regulated in the obese minipigs. An example is *LCN2* which is downregulated in the obese liver. In a study of human morbidly obese patients, *LCN2* is induced by inflammatory cytokines and is shown to be regulated by TNF-α [69]. LCN2 has target sites for miR-34a and miR-1285, which are both upregulated in the liver. MiR-34a is also upregulated in the liver of obese mice [70].

An explanation for the reverse expression direction of some obesity relevant genes could be that the Göttingen minipigs in this study represent a more healthy obese phenotype since they do not have significant differences in plasma lipids and insulin resistance [6]. The minipigs had access to *ad libitum* standard pig chow, with no excess fat and sugar contents as is seen in many studies of diet-induced obesity, including studies where female minipigs develop differences in plasma lipids [8]. Moreover, the obese minipigs used in the present study had previously been subjected to pharmacological studies of therapeutic peptides. Therefore, it cannot be ruled out, that the peptides might have permanently altered the gene expression towards a more healthy obese direction. However, the overall gene expression mimics what has been observed in other pig, human and rodent studies. Follow-up studies on obese minipigs fed a high fat diet versus a regular pig diet, on pigs that have not been subjected to previous experimental studies, would be highly relevant.

In conclusion, the expression pattern of a number of obesity-relevant genes and miRNAs in minipigs is in accordance with the expression pattern seen in comparable humans and rodent studies. On the other hand, the expression pattern of some of the transcripts investigated in is discordant with studies in humans and rodents, which could be a consequence of a slightly healthier obese phenotype of the Göttingen minipigs used in this study. Most importantly, many of the differentially expressed genes have target sites for miRNAs, and these miRNAs are expressed in the opposing direction, confirming the importance of miRNA-mediated regulation of genes in obesity.

## Supporting Information

S1 Dataset Raw Cq values and t-test result from qPCR. (XLSX)

S2 Dataset miRNA-target analysis. (XLSX)

S1 Table Primer sequences for miRNAs and mRNAs. (XLSX)

S1 Text List of abbreviations. (DOCX)
