# Supplementary material for: Joint Profiling of miRNAs and mRNAs Reveals miRNA Mediated Gene Regulation in the Göttingen Minipig Obesity Model
Source: PLoS One. 2016 Nov 30;11(11):e0167285. doi: 10.1371/journal.pone.0167285 (PMC5130236; doi:10.1371/journal.pone.0167285)
Supplement: S1 Text — (DOCX) [file pone.0167285.s004.docx]

Abbreviation List

ATP Adenosine Triphosphate

BLAST Basic local alignment search tool

cDNA complementary DNA

Cq Quantification Cycle

dATP deoxyAdenosine triphosphate

dCTP deoxycytidine triphosphate

dGTP deoxyguanosine triphosphate

dNTP deoxy nucleotide

dTTP deoxythymidine triphosphate

FASTA Fast-all

FC Fold change

miRNA microRNA

mRNA messenger RNA

ORF open reading frame

qPCR quantitative real-time polymerase chain reaction

QTL Quantitative trait loci

RT Reverse transcription

refSeq Reference sequence

UTR untranslated region

All gene names can be found in the primer table in supplementary file S1.
